# Transient and reversible focal lesion involving the splenium of the corpus callosum in a person with epilepsy

**DOI:** 10.4103/0972-2327.41883

**Published:** 2008

**Authors:** Nitin C. Parikh, Makarand Kulkarni

**Affiliations:** Department of Radiodiagnosis, Lilavati Hospital and Research Center, Bandra (W), Mumbai, India

## Case Report

A 40-year-old man with epilepsy was admitted with relapse of generalized tonic–clonic seizures following inadvertent discontinuation of phenytoin that he had been taking regularly. His neurological examination and previous brain magnetic resonance imaging (MRI) were normal. A repeat MRI (1.5-Tesla superconducting magnet, Symphony, Siemens, Germany) of the brain, using axial T1-weighted spin-echo (500 TR / 11 TE), axial and sagittal T2-weighted turbo spin-echo (6000 TR / 90 TE), axial FLAIR, and diffusion-weighted and ADC sequences, revealed a solitary well-defined ovoid lesion in the splenium of the corpus callosum, measuring 15 × 20 mm in size. The lesion was isointense to minimally hypointense on T1-weighted sequences, hyperintense on T2-weighted and FLAIR sequences, and showed restricted diffusion with low ADC values (40–50) [Figure 1a–c]. Axial, sagittal, and coronal T1-weighted sequences were obtained after intravenous injection of gadolinium (0.1 mmol/kg gadopentetate dimeglumine). Postcontrast MRI showed no significant enhancement of the lesion [Figure 1d]. The rest of the brain parenchyma was normal. The patient became asymptomatic after resuming phenytoin. An MRI of the brain, repeated after 4 weeks, revealed complete disappearance of the splenial lesion. He remained normal 6 months later.

**Figure 1(a):**
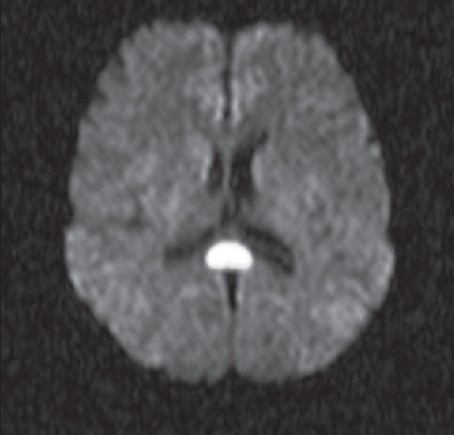
Axial diffusion-weighted MRI sequence shows a well-defined ovoid nodular lesion with restricted diffusion in the center of the splenium of the corpus callosum

**Figure 1(b):**
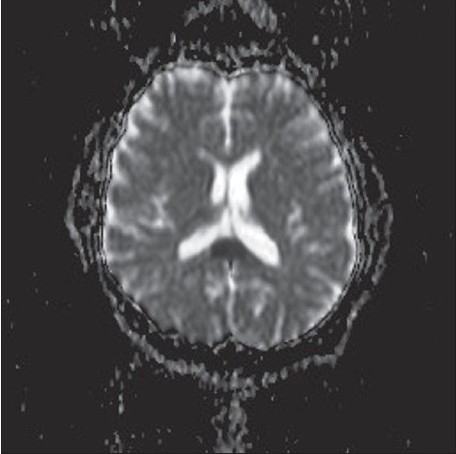
Axial ADC image shows that the lesion has a low ADC value (40–50)

**Figure 1(c):**
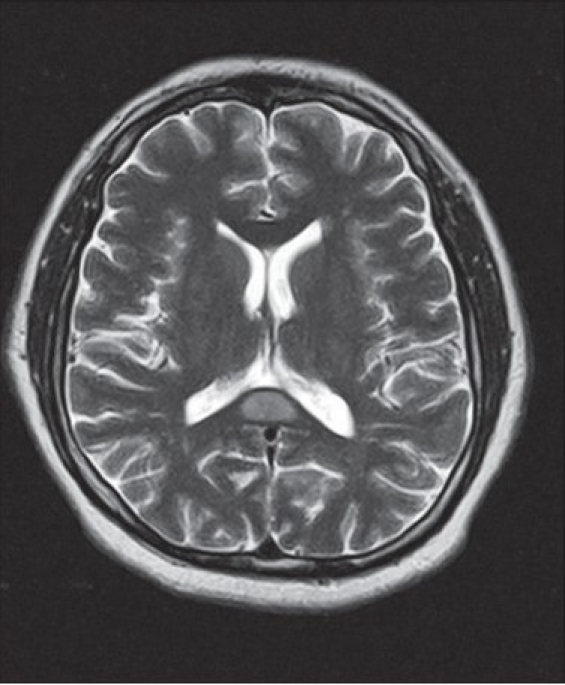
Axial T2-weighted sequence shows that the lesion is homogeneously hyperintense compared to the body of the corpus callosum

**Figure 1(d):**
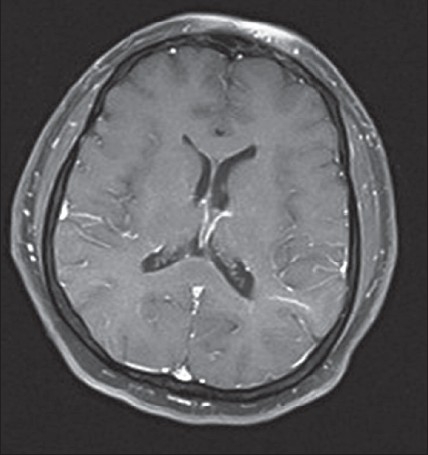
Axial T1W gadolinium-enhanced image shows that the lesion shows no significant enhancement

## Discussion

Discrete focal nonhemorrhagic lesions within the central portion of the splenium of the corpus callosum without any other accompanying lesion frequently pose a diagnostic dilemma for the clinician.[1] It is important to be aware that reversible focal lesions can occur rarely after seizures. Reversible MR signal changes in the splenium can occur due to vasogenic edema following a seizure,[1–3] withdrawal of an antiepileptic drug,[1,2,4–6] antiepileptic drug toxicity,[1] multiple sclerosis, trauma, infarct, neoplasm, adrenoleukodystrophy and other leukodystrophies, AIDS dementia complex, Marchiafava–Bignami disease,[1,7] or childhood-onset anorexia nervosa.[8] Reversible splenial signal changes due to vasogenic edema can occur in acute herpes simplex cerebellitis.[9,10] It is hypothesized that these signal changes may be related to alteration in the arginine–vasopressin system[3] or exitotoxic injury to astrocytes.[2,11]

A similar lesion was observed in a patient with an episode of kaleidoscopic vision while using diet pills containing sympathomimetic drugs[12]; withdrawal of the medication resulted in the cessation of the episodes and normalization of the MRI.
